# Stimuli-Responsive Soft Untethered Grippers for Drug Delivery and Robotic Surgery

**DOI:** 10.3389/fmech.2017.00007

**Published:** 2017-07-26

**Authors:** Arijit Ghosh, ChangKyu Yoon, Federico Ongaro, Stefano Scheggi, Florin M. Selaru, Sarthak Misra, David H. Gracias

**Affiliations:** 1 Chemical and Biomolecular Engineering, Johns Hopkins University, Baltimore, MD, United States; 2 Materials Science and Engineering, Johns Hopkins University, Baltimore, MD, United States; 3 Surgical Robotics Laboratory, Department of Biomechanical Engineering, MIRA - Institute for Biomedical Technology and Technical Medicine, University of Twente, Enschede, Netherlands; 4 Division of Gastroenterology and Hepatology, Department of Medicine, Johns Hopkins University, Baltimore, MD, United States; 5 Department of Biomedical Engineering, University of Groningen and University Medical Centre Groningen, Groningen, Netherlands

**Keywords:** robotics, surgery, computer-assisted, nanotechnology, microtechnology, polymers

## Abstract

Untethered microtools that can be precisely navigated into deep *in vivo* locations are important for clinical procedures pertinent to minimally invasive surgery and targeted drug delivery. In this mini-review, untethered soft grippers are discussed, with an emphasis on a class of autonomous stimuli-responsive gripping soft tools that can be used to excise tissues and release drugs in a controlled manner. The grippers are composed of polymers and hydrogels and are thus compliant to soft tissues. They can be navigated using magnetic fields and controlled by robotic path-planning strategies to carry out tasks like pick-and-place of microspheres and biological materials either with user assistance, or in a fully autonomous manner. It is envisioned that the use of these untethered soft grippers will translate from laboratory experiments to clinical scenarios and the challenges that need to be overcome to make this transition are discussed.

## INTRODUCTION

Biomedical applications, such as minimally invasive surgery (MIS) (Dogangil et al., 2010) or controlled and sustained drug delivery (Traverso and Langer, 2015) require new approaches in materials synthesis, fabrication, and robotic control. Present day MIS procedures utilize laparoscopic or catheter-based technologies in which a variety of tethered probes with imaging, suctioning, cutting, cauterizing, or suturing modalities are inserted through small external or internal incisions. These methods have significantly reduced invasiveness and patient trauma in many surgical procedures. A classic example is the minimally invasive mitral valve repair, which previously could only be achieved using the significantly more invasive bypass heart surgery (Felger et al., 2001). However, MIS procedures like intracranial stenosis (Lazzaro and Chen, 2010), video-assisted robotic thoracic surgery (Cerfolio et al., 2016), or ureteral hysterectomy (Packiam et al., 2016) performed with tethered probes in deep and/or tortuous regions of the body still suffer from compromised dexterity and inaccessibility, or risk of injury. The use of the tether or connection to external controls may also cause injuries due to motion of highly deformable soft tissues (Dogangil et al., 2010). Further, it can be challenging, if not impossible, to access submillimeter tortuous regions, or a highly branched system such as the capillary network in the vascular system.

New MIS techniques in which an untethered robotic surgical tool can be inserted and guided to a specific location to perform a surgical task in the body are emerging (Nelson et al., 2010; Sitti et al., 2015). These untethered devices have a negligible footprint to perform tasks like drilling, biopsy, small tumor ablation, as well as delivery of small molecule drugs or biomolecules to locations in previously hard to access regions in the body. Recent demonstrations include biopsies of the biliary tree of a live pig (Gultepe et al., 2013), delivery of a drug-simulant Rhodamine-B to the posterior segment of the eye in a rabbit (Fusco et al., 2014b), and patching wounds in *in vitro* gastrointestinal models (Miyashita et al., 2016). In addition to untethered operation, the use of materials that are compliant to tissues reduces the possibility of collateral injury during *in vivo* use, especially in hard to reach places (Majidi, 2014).

A variety of soft robot and, in particular, gripper actuation mechanisms have been developed and many of them have been envisioned for alternate applications, such as deep sea exploration (Galloway et al., 2016). The reader is directed elsewhere (Rus and Tolley, 2015) for a more comprehensive overview on the recent developments of soft robotic actuators and their applications. This mini-review describes soft robotic and, in particular, gripping tools that are suitable for biomedical applications. A broad discussion of soft-gripping actuation mechanisms is presented in the context of *in vivo* and clinical applicability. The review then focuses on a class of potentially clinically relevant stimuli-responsive soft-grippers (Malachowski et al., 2014; Breger et al., 2015), which can be controlled in an untethered manner and navigated to specific locations. These physiological stimuli-responsive grippers are made of polymers and hydrogels having a modulus in the range of 100 kPa to 200 MPa (Breger et al., 2015) and thus have a rigidity similar to biomaterials such as tissue (Rus and Tolley, 2015). The navigation and imaging techniques that have been developed to robotically maneuver these grippers is described thereafter. The review ends with a discussion of the developments that are required to take such soft gripping tools to clinical environments.

### SOFT GRIPPER ACTUATION MECHANISMS IN MEDICINE

Soft robotic actuation can provide enhanced capabilities for endoscope or catheter-based MIS procedures (Loeve et al., 2010; Mazzolai et al., 2012; Martinez et al., 2013; Cianchetti et al., 2014) by providing local and on-demand stiffening and better maneuverability. Also, due to their easily deformable shapes and larger degrees of freedom, the soft robots have the additional advantage of conforming to the shape and morphology of a surface in a passive manner resulting in a better contact (Hughes et al., 2016). This is evident in the universal gripper (Brown et al., 2010), which is based on the jamming transition of granular materials or the gecko feet-inspired gripper (Song and Sitti, 2014). A miniaturized soft gripper, which can conform to the shape of any soft material can lead to new ways to measure the stiffness of small tumors and more efficient drug delivery. Below, the most common soft gripper actuation mechanisms are classified. A comparative analysis of the different methods can be found in Table 1.

Electroactive polymers (EAPs) (Bar-cohen, 2004) have a sandwiched elastomer in between two metal electrodes. When a large voltage (~kilovolt to megavolt) is applied, the polymer can be bent by electrostatic interactions and has for example been used to fabricate a highly compliant soft gripper (Shintake et al., 2016) that can handle a wide variety of delicate materials like eggs and paper. Polymers like ionic polymer metal composites (Shahinpoor et al., 1998) or conjugated polymers (Smela, 2003), on the other hand, can operate at a significantly lower voltage (~volts) and still produce strains as large as EAPs. Macroscale gripping tools replicating the human hand (Polygerinos et al., 2015) have been used to assist limb movements of patients with compromised motor functions due to stroke or cerebral palsy.

More recently, pneumatic and fluidic actuators (Ching-Ping and Hannaford, 1996), which are based on utilizing a pressurized gas or liquid have been used to create a soft-robotic hand (Deimel and Brock, 2016). The robotic hand was able to grasp objects such as a telephone and a pair of chopsticks. A pneumatic network inside parallel inflatable elastomer chambers was used to generate complex motions of a gripper and grasp an anesthetized mouse (Ilievski et al., 2011).

Elastic materials like polyurethane and Sylgard have been mixed with powders of neodymium iron boride to make millimeter-sized magnetically actuated grippers that were actuated and navigated selectively with programmable and dynamic magnetization capabilities (Diller and Sitti, 2014; Zhang et al., 2017). Magnetic actuation has also been used to generate gripping actions in a miniaturized device made of highly elastic Nitinol alloy, which could be used to cut tissue from an *ex vivo* porcine liver (Ullrich et al., 2015).

The shape memory effect has been exploited in hard alloys of Ni–Ti to develop very thin catheters for natural orifice transluminal surgery (Chiang, 1988; Phee et al., 2002; Koh and Cho, 2013; Cianchetti et al., 2014), in which the heat-induced martensitic–austenitic phase transformation can result in peristaltic or inchworm like motion. Shape memory polymers (SMPs), on the other hand, can also transform shape to a predefined form when subjected to an appropriate stimulus such as temperature or light (Lendlein and Langer, 2002; Lendlein et al., 2005). They have been used to develop smart sutures or wound closures, for aneurysm treatment as well as to develop blood clot removal tools (Lendlein et al., 2010).

While the electrostatic/ionic and the fluidic actuation schemes require an electrical or fluidic connection to the outside world, the magnetic-or SMP-based actuators can be untethered. Another promising technique for generating untethered actuation at small length scales are stimuli-responsive hydrogels and polymers (de Las Heras Alarcon et al., 2005b; Andersen et al., 2009; Ionov, 2011, 2013; Gracias, 2013). For example, when two layers of polymers having different swelling ratios form a bilayer, then swelling can trigger bending (Hu et al., 1995; Kwag et al., 2016). Unlike SMPs, these stimuli-responsive polymers and hydrogels can be readily patterned and photocrosslinked using a variety of techniques including conventional photolithography and even 3D printing (Gladman et al., 2016) giving rise to a variety of shape changing structures that could be utilized in a range of clinical scenarios.

### DESIGN, FABRICATION, AND ACTUATION OF STIMULI-RESPONSIVE SOFT GRIPPERS

**FIGURE 1 |:**
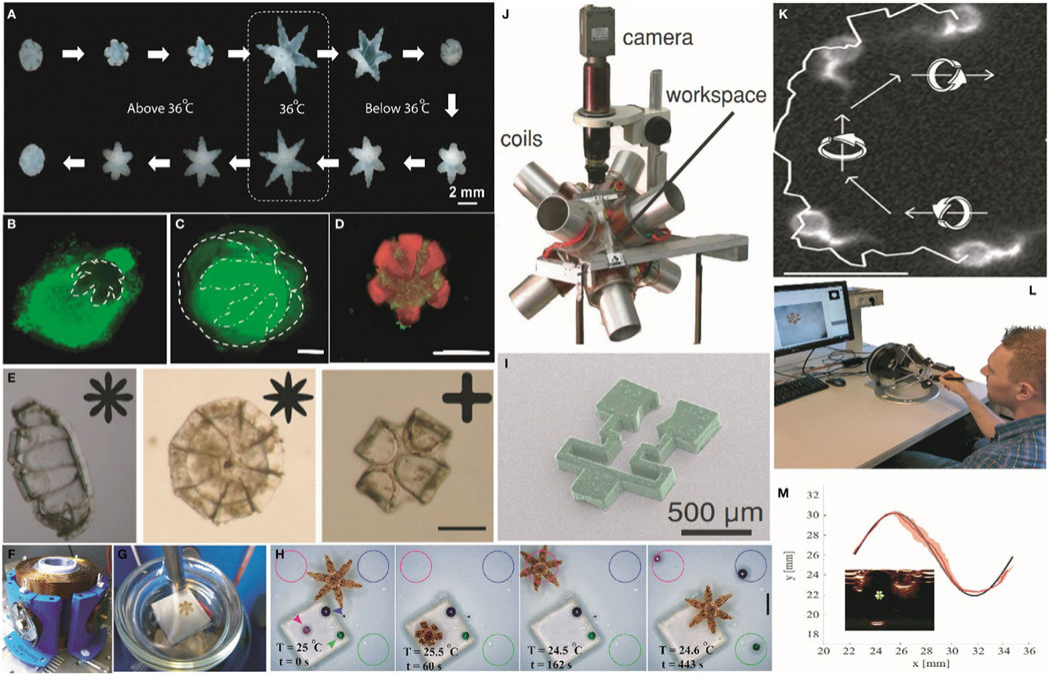
Actuation, navigation, and tracking of untethered stimuli-responsive soft grippers. **(A)** Experimental snapshots showing the actuation of a soft stimuli-responsive gripper in response to heating and cooling above and below physiological temperature. Reprinted with permission from Breger et al. (2015) ©ACS Publications. **(B)** Capture and excision of a lump of fibroblast cells (scale bar = 1 mm). Reprinted with permission from Breger et al. (2015) ©ACS Publications. **(C)** A gripper with the excised lump of fibroblast cells in its grasp (scale bar = 500 μm). Reprinted with permission from Breger et al. (2015) ©ACS Publications. **(D)** A soft gripper eluting chemotherapeutic drug doxorubicin while grabbing a clump of breast cancer cells (scale bar = 1 mm). Reprinted with permission from Malachowski et al. (2014) ©John Wiley and Sons. **(E)** IR-responsive self-folding soft grippers fabricated from PEGDA and a composite of graphene oxide and pNIPAm. Scale bars = 200 μm. Reprinted with permission from Fusco et al. (2014a) ©2013, WILEY-VCH Verlag GmbH and Co. KGaA, Weinheim. **(F)** An electromagnetic coil setup used to navigate the soft grippers. Reprinted with permission from Ongaro et al. (2016b) ©2016, IEEE. **(G)** A soft gripper on a Petri dish as mounted in the magnetic coil. The white background is the Peltier element used to heat the gripper. Reprinted with permission from Ongaro et al. (2016b) ©2016, IEEE. **(H)** A fully autonomous object sorting task executed by a thermoresponsive magnetic gripper, in which differently colored beads were picked up and placed in the similarly colored circle. The second and the third images in the sequence show the detailed sorting of the pink colored bead. Scale bar = 2 mm. Reprinted with permission from Ongaro et al. (2017) ©2016. **(I)** A magnetic gripper containing two different materials, which can be both actuated and navigated using a **(J)** magnetic coil setup. Reprinted with permission from Diller and Sitti (2014) ©2014, WILEY-VCH Verlag GmbH and Co. KGaA, Weinheim. **(K)** The smallest soft microrobot till date that exploits bundle of assembled DNA for generating swimming-based propulsion using a rotating magnetic field. Scale bar = 10 μm. Reprinted with permission from Maier et al. (2016) ©2016, American Chemical Soceity. **(L)** The haptic interface used for controlled navigation of stimuli-responsive soft grippers by human users. Reprinted with permission from Pacchierotti et al. (2017) ©1969, IEEE. **(M)** Motion of a soft gripper in a sinusoidal path using ultrasound image feedback. The SD in tracking the gripper is denoted by the red shaded area. Inset, an ultrasound image of the gripper (Scheggi et al., 2017, ©2017, IEEE).

As noted above, stimuli-responsive materials are attractive for designing robots with untethered actuation. The use of temperature as the stimulus ensures proper functionality independent of the chemical composition of the environment. Though there are many thermoresponsive polymers (Gil and Hudson, 2004), in order to make the actuation of the soft robotic gripper fully autonomous at physiological temperatures, the thermally responsive polymer pNIPAm (Hirokawa et al., 1984; Schild, 1992; Schmaljohann, 2006) is an appropriate choice. The co-polymer system of pNIPAm and acrylic acid shows a sharp transition from hydrophilic to hydrophobic states in the tunable range of physiological relevance between 32 and 38°C. Breger et al. (2015) used this property to engineer soft microgrippers, which can be triggered to open and close autonomously, using physiological temperature as the stimulus (Figure 1A), with verified reversibility over 50 thermal cycles. However, pNIPAm is a very soft, deformable material and needed to be integrated with a significantly stiffer material like poly-propylene fumarate or SU8 photoresist to apply a secure grip. The inclusion of a stiff segment enabled applicability to excision of soft tissue as illustrated in Figures 1B,C, where a gripper excised a clump of cells from a cell culture mass. Breger et al. (2015) also implemented a finite element model to simulate the effect of various design parameters on the opening and closing of a soft gripper based on the balance between entropic and enthalpic interactions and the mechanical forces of swelling. Such models can be used to tune parameters such as layer thickness and swelling ratios needed for optimal performance.

In addition, soft grippers, which are composed of polymer and hydrogel layers can be made porous, and thus can also be loaded with drugs (Gupta et al., 2002; Kikuchi and Okano, 2002; Xia and Pack, 2015). For example, Malachowski et al. (2014) loaded soft grippers with anti-inflammatory and chemotherapeutic drugs like mesalamine and doxorubicin (Figure 1D) using different methods to achieve drug release over different temporal periods. The therapeutic grippers or theragrippers were small enough to be deployed using endoscopic catheters under *in vivo* conditions, and they were used to elute a dye in the stomach of a live pig. The successful loading and release of drugs in a controlled manner from the theragrippers offers the possibility for the realization of self-gripping drug delivery patches that could potentially grab onto tissue and release drugs for an extended period of time. Such chemomechanical devices are attractive because they could augment patches that rely only on mucoadhesives (Andrews et al., 2009).

It should be noted that composites of pNIPAm can respond to alternate stimuli such as pH, light, and ionic strength. For example, devices composed of pNIPAm, graphene, and pEGDA composites (Fusco et al., 2014a) have been developed (Figure 1E) where the gripping action was triggered by near infrared radiation (NIR). The choice of material is not unique to pNIPAm and other soft materials could also be utilized. Thus, IR wavelength selective bending has also been achieved using liquid crystalline elastomers (Kohlmeyer and Chen, 2013). Instead of using physiological temperature as the stimulus, pH-responsive hydrogels can also be utilized. These include patterned bilayers of PMMA and poly (EGDMA) (Guan et al., 2005) or poly (HEMA-co-AA) and poly (HEMA) (Shim et al., 2012), which swell differently under acidic and basic conditions. For example, Li et al. (2016) demonstrated the fabrication of a gripper from a bilayer of pHEMA and pEGDA, in which the grippers were closed at higher pH and opened at lower pH releasing microbeads. It is noteworthy that care must be taken to passivate or coat the surfaces of the robots to avoid biofouling, clotting, or infection, especially when they are used over longer periods of time such as for drug delivery. Non-fouling agents such as polyethylene glycol, poly (2-hydroxyethyl methacrylate), as well as zwit-terionic hydrogels have been developed (Castner and Ratner, 2002; Zhang et al., 2013) to reduce interactions between the soft-device and the body and minimize trauma, or side effects. In addition, polymers like pNIPAm has been shown to attach to cells and bacteria in a temperature-dependent manner (de Las Heras Alarcon et al., 2005a; Cooperstein and Canavan, 2010; Schmidt et al., 2010), which can lead to unwanted accumulation of cells on the robots.

### NAVIGATION AND TRACKING OF UNTETHERED STIMULI-RESPONSIVE GRIPPERS: TOWARD TARGETED THERAPY

Though autonomous and untethered actuation of the grippers allow them to be safely manipulated inside a live animal, it is important to navigate and control their spatial position to carry out tasks in specific locations. Untethered navigation of soft robots is an emerging field of research with only few demonstrations of successful locomotion. SMAs and dielectric elastomers have been used to generate caterpillar or inchworm like locomotion (Seok et al., 2010; Lin et al., 2011). Fluid pressurization and depressurization have led to a quadrupedal multigait robot capable of locomotion underneath obstacles (Shepherd et al., 2011). A centimeter scale battery-operated soft robotic fish was shown to exhibit swimming motion under water (Marchese et al., 2014).

The above examples of navigation that exploit the flexibility of the soft robotic body often require a surface to assist motion and require a tether/battery for power delivery. While these schemes might be useful to operate in larger spaces, the realization of tether less navigation in a millimeter or submillimeter scale is difficult because of the inherent complexity of the fabrication. In order to actuate a multifunctional miniaturized surgical gripper, alternate schemes need to be explored, so that they can be easily implemented in a clinical scenario. A variety of methods have been developed to achieve tetherless navigation of miniaturized agents. Chemical motors, where the surrounding environment provides the fuel, which reacts with the micromotor to generate thrust, have been used to propel cargo and achieve functional tasks (Paxton et al., 2004; Mano and Heller, 2005). Other sources of energy like electricity (Calvo-Marzal et al., 2010), light (Eelkema et al., 2006), or ultrasound (US) (Wang et al., 2012; Garcia-Gradilla et al., 2013) have also been used to transport microscale spheres and bacteria-like *E. coli* or *S. aureus.* In a particularly attractive demonstration, structured monochromatic light was used to achieve locomotion in a millimeter scale robot made of liquid crystal elastomers (Palagi et al., 2016). However, these schemes either depend on the composition of the surrounding media, cannot propagate through tissue, or can be difficult to implement *in vivo* because of high power requirements, three dimensionality, and the presence of obstacles.

In contrast, magnetic fields have been shown to be very suitable for generating propulsion (Zhang et al., 2009; Ghosh et al., 2012) or to provide directionality (Ghosh et al., 2014) in various materials ranging from biofluids like human blood (Lekshmy Venugopalan et al., 2014) to the peritoneal cavity of a live mouse (Servant et al., 2015). They pose very minimal risk of injury and can be transmitted through both opaque and transparent objects. Previously, a millimeter-sized particle of chrome-steel was shown to be safely manipulated inside the carotid artery of a live pig (Martel et al., 2007) using gradient magnetic fields generated by a magnetic resonance (MR) imaging machine. Untethered magnetic actuation of soft grippers can be achieved by impregnating magnetic particles in the body of the robot. Ongaro et al. (2016a,b, 2017) used gradient magnetic fields (Figures 1F,G) along with visual and control algorithms to navigate soft stimuli-responsive grippers on a piece of porcine tissue and avoid both static and dynamic obstacles. The closed loop system even allowed the grippers to perform autonomous pick and place tasks (Figure 1H) with squishy biological materials like egg yolk. Alternatively, human operator-assisted guidance could also be enabled (Ongaro et al., 2016a; Pacchierotti et al., 2017), such as with a haptic feedback control, which is envisioned in a surgeon-assisted intervention (Figure 1L). Similarly, millimeter scale soft magnetic grippers were loaded with neodymium iron boride particles to achieve navigation (Figures 1I,J) in a spatially selective manner (Diller and Sitti, 2014). Other examples of magnetic actuation of miniaturized soft robots include swimming bundles of DNA (Figure 1K) (Maier et al., 2016) and pNIPAm-based shape changing swimmers (Huang et al., 2016). In addition, magnetic fields were used to navigate the motion of small scale bio-hybrid robots like magnetosperms (Magdanz et al., 2013) or magnetotactic (Khalil et al., 2013) bacteria and were used to carry drugs to tumor hypoxic regions (Felfoul et al., 2016).

It is noteworthy that since the body is opaque to visible light, it can be challenging to enable vision-based feedback and tracking. Consequently, feedback based on US images has also been demonstrated (Scheggi et al., 2017), as US provides sufficient depth of imaging inside the body with millimeter scale resolution, apart from being inexpensive. Coupled with a magnetic motion control system, US imaging was used for automated pick and place with soft grippers (Figure 1M).

### CHALLENGES FOR *IN VIVO* TRANSLATION AND FUTURE DIRECTIONS

Biologically inspired robotics have led to the embodiment of different locomotion and sensing capabilities including robots with self-learning capabilities (Pfeifer et al., 2007; McEvoy and Correll, 2015). While exciting, many of these robots require an external power source; but, for tasks like targeted *in vivo* surgery, a tether can seriously limit the region of applicability. Integrating an adequately powered source of electrical/pneumatic energy on to the body of a miniaturized functional soft robot is still a challenge. As highlighted, the stimuli-responsive soft grippers described here overcome this limitation. However, several challenges need to be overcome to enable translation of these devices to the patient.

Innovative solutions are required in materials synthesis and soft-robot integration, to perform tasks in complex, cluttered, and dynamic *in vivo* environments. For example, the soft grippers, described here, actuate within a few minutes of being exposed to the body temperature when inserted from a cold state. But for some applications like targeted surgery or delivery, the triggering mechanism necessitates that the grippers close only when they reach the target site, which may occur at shorter or longer times. Hence, it is necessary to develop grippers that can respond to other physicochemical cues such as pH (Guan et al., 2005; Shim et al., 2012), enzymes, or other biomolecular triggers (Bassik et al., 2010), or external optical and magnetic stimuli (Zrinyi, 1997; Zhang et al., 2011).

In order to fully realize the vision of untethered surgery, other tool designs such as cutters, tweezers, or microscopic sutures also need to be developed. In order to successfully execute such tasks, material properties like stiffness or morphology of the shapes have to be taken into account so that sufficient forces are exerted to carry out the tasks (Hughes et al., 2016). Fabricating these complicated devices using 2D microfabrication schemes are challenging and will require new fabrication techniques like 3D printing or two photon polymerization (Maruo et al., 1997; Rengier et al., 2010).

An important focus area is patient safety; in the absence of a tether, there is always the risk that the robot could get lost or lodged within tissue and hence it is preferred if the tools are composed of biodegradable materials so that they can be broken down and cleared from the body. It should be noted that there are a number of soft biodegradable materials that have been developed, primarily for tissue engineering applications and these could be utilized in the synthesis of dissolvable soft robots (Hutmacher et al., 2001; Gunatillake and Adhikari, 2003).

In terms of tracking, further studies using US, MR, or NIR need to be done in animal models to demonstrate feasibility. Though MR shows very good penetration in the deep tissues, it suffers from poor speed (~minutes) to achieve a high enough resolution (≈400 μm) (Bodle et al., 2013; Van Veluw et al., 2013). These problems are partially solved with techniques like magnetic particle imaging (MPI) (Weizenecker et al., 2009), where a resolution of around 400 μm can be achieved with a temporal resolution as low as 21 ms. MPI has been shown to be able to image the beating heart of a live mouse. As described in the previous section, US imaging can provide almost real-time imaging but still suffers from limited spatial resolution (Wells, 2000; Nelson et al., 2010). NIR or radiolabeling using quantum dots or nanoparticles on the other hand, works well only very close to the surface of the skin as it quickly attenuates inside the body (Frangioni, 2003; Gao et al., 2004) and thus imaging very small robots deep inside the body is still a challenge and breakthroughs in imaging technology are needed.

As discussed above, soft robots have been used to perform drug delivery or surgery in the GI tract of large animals. However, to extend their use in more confined spaces like the vascular system, they need to be further miniaturized. To achieve navigation in blood vessels that are 20–30 μm in width or even smaller, stronger magnetic materials like Fe–Co or Ni–Co alloys need to be investigated (Pouponneau et al., 2009). Inspiration for more effective transport *in vivo* can also be obtained from nature where one/group of flexible flagellum/flagella can either be rotated in a helical fashion (Ghosh and Fischer, 2009) or waved in a single plane (Maier et al., 2016) to generate propulsive force in bacterial systems.

In conclusion, miniaturized untethered soft robotic structures that can be navigated through tortuous paths can deliver drugs or carry out surgical tasks in locations that are impossible to reach non-invasively using current tethered devices. The field is in its infancy and stimuli-responsive soft robots could benefit from recent advances in stretchable electronics and onboard energy harvesting (Bauer et al., 2014; Lu and Kim, 2014), to broaden applicability by enabling sensing and communication to outside world (McEvoy and Correll, 2015). With the collaborative effort of scientists, engineers, and doctors, smart *in vivo* communicable soft untethered robots that can perform on demand therapy might not be a distant dream.

## Figures and Tables

**TABLE 1 |:** Classification of the common actuation mechanisms for soft biomedical robots/grippers.

actuation mechanism	Advantages	Drawbacks	Reference

Electrostatic/ionic	•Precise control of local motion •Reproducible response •Reversible response	•Needs a tether •Often requires high voltage •Concerns with electrical burns •Challenging to miniaturize •Slow response •Short lifetime	Polygerinos et al. (2015), Shintake et al. (2016)

Pneumatic/fluidic	•Precise control of local motion •Safe for *in vivo* use •Insensitive to the environment •Complex actuation possible	•Needs tubing •Needs external gas, fluid, compressor •Challenging to miniaturize •Challenges with fittings, burst failure	Ilievski et al. (2011), Mazzolai et al. (2012), Martinez et al. (2013), Deimel and Brock (2016)

Magnetic	•Untethered Actuation •Can be miniaturized •Independent of composition of the environment	•Actuation setup can be very complicated •Large impractical 3D magnetic fields and gradients may be required to actuate very small structures in humans	Diller and Sitti (2014), Ullrich et al. (2015), Zhang et al. (2017)

Shape memory materials	•Untethered actuation •Safe for *in vivo* use •Fast actuation •Independent of composition of the environment	•Irreversible actuation •In most cases, temperature is the only stimulus, which can limit applicability •Pre-deformation often required •Challenging to miniaturize	Lendlein and Langer (2002), Lendlein et al. (2010), Koh and Cho (2013)

Stimuli responsive polymers	•Untethered actuation •Extreme miniaturization possible •Autonomous •Ease of manufacturability; can be 3D printed	•Typically slow •Dependent on the composition of the environment	Fusco et al. (2014a), Malachowski et al. (2014), Breger et al. (2015)
